# Cholecystectomy in the Context of Cirrhosis, Sclero-Atrophic Cholecystitis, and Gangrenous Cholecystitis: A Literature Review

**DOI:** 10.3390/medicina61081314

**Published:** 2025-07-22

**Authors:** Cristian Botezatu, Dumitru Dragos Chitca, Valentin Popescu, Martina Nichilo, Angela Madalina Lazar, Bogdan Mastalier

**Affiliations:** 1 General Surgery Department, Carol Davila University of Medicine and Pharmacy, 8 Eroii Sanitari Blvd., 050474 Bucharest, Romania; cristian.botezatu@umfcd.ro (C.B.); valentin.popescu@drd.umfcd.ro (V.P.); angela.lazar@umfcd.ro (A.M.L.); bogdanmastalier@yahoo.com (B.M.); 2 General Surgery Clinic, Colentina Clinical Hospital, 020125 Bucharest, Romania; martina.nichilo@rez.umfcd.ro

**Keywords:** cholecystectomy, liver cirrhosis, sclero-atrophic cholecystitis, acute gangrenous cholecystitis

## Abstract

The gallbladder pathology is mainly represented by cholelithiasis, treated with cholecystectomy, one of the most commonly performed surgical procedures, continues to raise some challenges. Despite the advancements in surgical techniques, especially in those patients presenting some particularities, such as cirrhotic patients or those with sclero-atrophic or acute gangrenous cholecystitis, difficulties continue to arise. This review, including an evaluation of the literature from the last 20 years, aims to explore the pathophysiological mechanisms and surgical approaches for these high-risk conditions. Emphasis is placed on tailoring management strategies in order to reduce complications and improve outcomes, offering insights for optimizing care in difficult cholecystectomies.

## 1. Introduction

Cholecystectomy, conducted more than 1.2 million times per year in the United States, is fundamental in the treatment of gallbladder disease [1]. Despite breakthroughs in surgical techniques enhancing results, problems remain in complex patients, resulting in elevated complication rates, extended hospital stays, and greater healthcare expenditures [2]. Complications such as bile duct damage, surgical infections, and hemorrhage present considerable concerns to patient safety and impose large economic burdens on global healthcare systems [3,4].

This review aims to explore the factors contributing to difficult cholecystectomies, focusing on three major conditions—cirrhosis, sclero-atrophic cholecystitis (SAC), and gangrenous cholecystitis (GC)—that heighten the risk of intraoperative and postoperative complications. By examining the pathophysiology, surgical challenges, and outcomes associated with these conditions, this article seeks to provide a comprehensive understanding of strategies to mitigate risks and improve surgical success.

## 2. Materials and Methods

This narrative review was conducted to summarize the current understanding and surgical approaches to cholecystectomy in complex clinical contexts, namely, liver cirrhosis, SAC, and GC.

A comprehensive literature search was performed using PubMed and Google Scholar for studies published between January 2005 and May 2024. The search strategy included combinations of keywords and MeSH terms: “cholecystectomy”, “liver cirrhosis”, “sclero-atrophic cholecystitis”, “gangrenous cholecystitis”, “laparoscopic surgery”, and “robotic surgery”.

The selection process involved two independent reviewers who screened titles and abstracts, followed by full-text evaluation. Discrepancies were resolved by consensus. References of included articles were manually reviewed to identify additional relevant sources.

Articles were assessed for their relevance to surgical indications, intraoperative challenges, perioperative management, and patient outcomes. The review was conducted in accordance with the SANRA (Scale for the Assessment of Narrative Review Articles) guidelines to ensure scientific quality.

## 3. Cholecystectomy in Cirrhosis

### 3.1. Pathophysiological Challenges of Cholecystectomy in Cirrhosis

Gallstones, particularly those in the gallbladder, are prevalent among patients with cirrhosis, with an incidence of 9.5–29.4%, in contrast to 5.2–12.8% in those without cirrhosis [5]. Their occurrence is increasing with age, female gender, thickness of the gallbladder wall, and severity of concomitant liver disease [6,7].

Liver cirrhosis introduces a unique set of pathophysiological challenges that complicate gallbladder surgery. The altered hepatic architecture and impaired liver function significantly increase surgical risks, requiring meticulous perioperative management.

Cirrhosis impairs gallbladder mobility and alters bile composition, frequently leading to gallstone development. Moreover, portal hypertension often results in gallbladder wall thickening, edema, and vascular engorgement, which may obscure anatomical landmarks during surgery, complicating dissection and heightening the risk of damage [8].

Patients with cirrhosis face heightened risks during cholecystectomy due to several key factors.

Cirrhosis diminishes the synthesis of clotting factors, leading to coagulopathy. This, together with thrombocytopenia due to splenic sequestration, elevates the risk of perioperative hemorrhage [9].

Increased portal venous pressure may lead to the formation of varices within the hepatobiliary vascular system, which carry a risk of rupture during surgical procedures. Moreover, this condition intensifies fluid shifts, resulting in perioperative instability [10].

Ascites presents challenges in the surgical field, heightening the likelihood of infection and hindering the process of wound healing. Persistent ascites correlates with unfavorable postoperative outcomes and an increased likelihood of hernia development [11].

Cirrhotic patients frequently exhibit immunocompromise, rendering them more vulnerable to perioperative infections, such as bile peritonitis in instances of gallbladder perforation [12].

### 3.2. Indications and Timing of Surgery: Elective vs. Emergency Cholecystectomy in Cirrhotic Patients

The timing of cholecystectomy in cirrhotic patients, whether elective or emergent, is a crucial factor influencing results. Elective cholecystectomy is typically advised for patients with compensated cirrhosis (Child-Pugh class A or B) as it facilitates preoperative optimization and diminishes the likelihood of perioperative complications. Conversely, emergency cholecystectomy is frequently required for acute illnesses such as gallbladder cancer or perforation, but it is associated with markedly elevated morbidity and fatality rates due to the patients’ impaired physiological status [13].

N’Guyen et al. conducted the following pre-operative recommendations [14,15]:

(a) Medically optimize the patient: manage ascites, rectify coagulopathy [administer fresh frozen plasma (when INR > 1.5) and platelets (<50/mm^3^)];
(b) Cirrhotic individuals with hemoglobin levels below 10 g/dL should get corrective blood transfusions prior to abdominal surgery;
(c) Acquire pre-operative imaging to identify abdominal wall varices or a recanalized umbilical vein and to exclude hepatoma [16]. Evaluate the use of a cholecystostomy tube in patients classified as Child class C (MELD > 13).

In addition to N’Guyen et al.’s recommendations [14,15]., preoperative serum albumin has emerged as a powerful independent predictor of postoperative morbidity and mortality in cirrhotic patients undergoing cholecystectomy. In a study by Lalhruaizela et al., patients with serum albumin levels below 3.0 g/dL experienced significantly higher rates of early and late postoperative complications, with a complication rate of 100% observed in those with albumin < 2 g/dL [17]. Vincent et al. further reported that albumin levels < 2.0 g/dL were linked with nearly 100% mortality in critically ill patients, independent of nutritional status and inflammation [18]. We conclude that a preoperative albumin correction better helps the patient’s recovery.

Frailty is a key predictor of postoperative complications. A study using NSQIP data (2005–2010) found that patients with intermediate and high frailty—measured by the Modified Frailty Index (mFI)—had significantly increased risks of major complications (OR up to 4.59) and mortality (OR up to 12.2) after LC for acute cholecystitis. The mFI demonstrated strong predictive accuracy, with an AUC of 0.83 for mortality. These findings emphasize the importance of frailty assessment in preoperative risk stratification and surgical planning for high-risk patients [19].

Elective laparoscopic cholecystectomy (LC) is preferred for cirrhotic patients who do not exhibit substantial portal hypertension or ascites. This method facilitates the optimization of coagulopathy and the control of comorbidities, thereby diminishing the risk of hemorrhage, bile duct damage, and postoperative infections [15]. Research indicates that elective operations yield reduced rates of conversion to open surgery and enhanced long-term outcomes relative to emergency interventions [8].

Standard coagulation tests like INR and aPTT often show abnormalities but do not accurately reflect bleeding risk due to a rebalanced hemostatic system. Although thrombocytopenia and prolonged PT are common, compensatory mechanisms such as elevated von Willebrand factor levels help maintain overall coagulation balance. Routine prophylactic correction of INR or platelet counts with FFP, vitamin K, or platelet transfusions has not shown benefit and may even increase risks, including volume overload and transfusion-related complications. Clinical evidence supports a “wait-and-see” approach, reserving correction only for active bleeding rather than relying on abnormal lab values alone. Advanced assessments, such as thromboelastography or thrombin generation testing, may provide a more accurate picture of coagulation status in cirrhotics, though they are not yet widely adopted [20].

In cirrhotic individuals, emergency cholecystectomy is frequently imperative in instances of severe acute cholecystitis or complications like perforation. Nonetheless, these treatments include increased risks of intraoperative hemorrhage, hemodynamic instability, and extended hospitalizations due to insufficient time for preoperative preparation [21]. The mortality rate for emergency procedures is significantly elevated, especially in patients with decompensated cirrhosis [22].

Determining the time of surgery necessitates a thorough evaluation of liver function, the extent of cirrhosis, and the immediacy of the clinical situation. In patients with advanced cirrhosis (Child-Pugh class C), non-surgical interventions like percutaneous transhepatic gallbladder drainage (PTGBD) may be contemplated as a temporary measure prior to elective surgery or final treatment [23,24].

### 3.3. Surgical Techniques and Modifications: Laparoscopic vs. Open Approaches and Robotic Innovations

The technical challenges associated with cholecystectomy in cirrhotic patients are numerous and can be categorized as follows:

1. Risk of collateral wound formation in the periumbilical region during optical trocar insertion;
2. Hemorrhagic risk associated with vascular adhesions;
3. Challenges in liver retraction and exposure of the Calot triangle;
4. Hazardous approach to the vesicular pedicle in the context of portal hypertension;
5. Hemorrhagic dissection of the vesicular bed. The challenges are further intensified in patients who have undergone surgery for acute or chronic cholecystitis [25].

Laparoscopic cholecystectomy (LC) has predominantly supplanted open cholecystectomy (OC) as the favored method for gallbladder excision, especially in patients with cirrhosis, owing to its reduced morbidity, abbreviated hospitalizations, and expedited recovery periods [26].

In cirrhotic patients, LC has the additional benefit of diminished ascites-related complications and reduced interference with hepatic blood flow compared to open cholecystectomy [8].

Nonetheless, obstacles persist with LC in this patient demographic, especially owing to the fragile tissue composition, altered anatomy resulting from portal hypertension, and the potential for hemorrhage from engorged veins inside the hepatobiliary triangle. In instances of advanced cirrhosis (Child-Pugh class C) or significant ascites, oral contraceptives may still be warranted, however, accompanied by increased risks [27].

**Figure 1:**
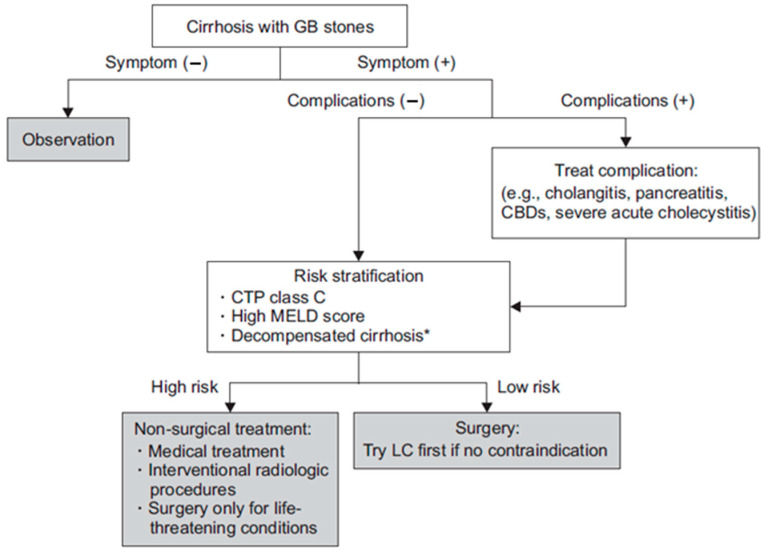
Wang et al.’s proposed flowchart for the management of cirrhotic patients with cholelithiasis [8]. * Decompensated cirrhosis: ascites and/or variceal bleeding occurrence.

Preoperative optimization, encompassing the correction of coagulopathy, management of ascites, and meticulous identification of patients with compensated cirrhosis, is essential for reducing surgical risks (Figure 1) [28].

Numerous operational details hold significance:

1. Surgical configuration: Although it is acknowledged that trocars disrupt collateral circulation to a lesser extent than a midline incision, the periumbilical region should be circumvented;
2. Following the insertion of the initial trocar, transillumination of the abdominal wall facilitates the avoidance of collateral structures;
3. The positioning of the subxiphoid port to the right of the midline prevents damage to the falciform ligament and the possibly repermeabilized umbilical vein;
4. If the left lobe intrudes upon the operational field, the surgeon should elevate the patient’s right shoulder and/or utilize a long port or converter placed into the epigastric port; if this is inadequate, an additional port should be inserted to retract the left lobe;
5. During the operation, it is crucial to prevent excessive traction on the gallbladder to avert avulsion from the liver bed;
6. Finally, in the context of other procedures in cirrhotic patients, particularly those with ascites, the use of intraperitoneal drains should be eschewed [29].

A 2020 study indicates that conversion to laparotomy occurs in 12.3% of individuals, with rates ranging from 0% to 20.8%. The predominant causes (Table 1) included vesicular bed hemorrhage in 41.3% of patients, inadequate visualization of structures in 26.1%, the presence of adhesions in 13.0%, excessive inflammation precluding safe laparoscopic surgery in 6.5%, and other causes in 13.0% of patients [5].

Robotic-assisted cholecystectomy (RC) provides superior dexterity, vision, and precision, rendering it especially beneficial in instances of anatomical distortion or vascular problems. Research comparing robotic-assisted surgery to laparoscopic procedures indicates similar or superior outcomes, including reduced operating durations and diminished conversion rates [26,30]. However, the expense and significant learning curve continue to impede wider usage [31].

### 3.4. Subtotal Cholecystectomy

Subtotal cholecystectomy (SC) is acknowledged as a safe alternative to total cholecystectomy; however, the long-term outcomes for patients are influenced by the existence of residual stones in the gallbladder remains. The retention rate of stones ranges from 4% to 15% [32,33]. While the majority of residual stones may be extracted using ERCP, some patients still have to undergo reoperations [34,35]. The incidence of long-term problems following SC is greater than that following total cholecystectomy. For individuals who can exclusively undergo SC because of the local situation of the hepatocystic triangle, laparoscopic surgery yields superior outcomes compared to the open technique [32].

Residual gallbladder stones after SC, particularly subtotal procedures, are a clinically significant complication that may cause recurrent symptoms such as pain, jaundice, or cholangitis. Missed or retained stones are often discovered months or years later, necessitating further interventions like ERCP, reoperation, or stone extraction techniques. Early identification through imaging modalities such as ultrasound or MRCP is critical in symptomatic patients with a history of cholecystectomy. Preventive strategies—including complete gallbladder excision where feasible and intraoperative inspection—are essential to reduce postoperative morbidity associated with retained stones [36].

While SC is increasingly recognized as a valuable salvage approach in complex biliary surgery, several authors have discussed the evolving nomenclature and technical variations of subtotal cholecystectomy, emphasizing the need for clarity in describing surgical intent, anatomical preservation, and anticipated outcomes [32]. The incidence of retained stone recurrence following SC is said to be about 1.8% according to Elshaer et al. [32].

Fenestrating SC was associated with a significantly higher incidence of postoperative bile leakage compared to the reconstituting approach (18% vs. 7%; *p* < 0.022). However, over a median follow-up period of six years (IQR: 5–10 years), patients who underwent fenestrating STC experienced a significantly lower recurrence of biliary events than those who received the reconstituting technique (9% vs. 18%; *p* < 0.022) [35].

In a retrospective cohort of 57 patients with a mean follow-up duration of 49 months, 5.3% developed symptomatic choledocholithiasis, 12.3% experienced incisional hernia, and 7% presented with symptomatic gallstones in the remnant gallbladder tissue within the first postoperative year [37].

Early detection of complications requires structured follow-up; in the immediate postoperative period, close clinical monitoring and liver function testing are essential to identify occult bile leaks or early signs of biliary obstruction [38].

## 4. Cholecystectomy in Sclero-Atrophic Cholecystitis

### 4.1. Etiopathogenesis of Sclero-Atrophic Cholecystitis

Sclero-atrophic cholecystitis (SAC) is the terminal phase of chronic gallbladder inflammation, marked by extensive fibrosis, atrophy of the gallbladder wall, and occlusion of the lumen. This process arises from chronic irritation, primarily caused by gallstones or biliary sludge, which triggers and sustains the inflammatory cascade [39].

The pathophysiology of SAC initiates with episodes of acute cholecystitis resulting from gallstones clogging the cystic duct. Repeated inflammatory episodes result in chronic cholecystitis, characterized by histological alterations including lymphocytic infiltration, glandular atrophy, and fibrosis [40].

With time, the gallbladder becomes inflexible and functionally dormant, increasing the risk of problems such as secondary bacterial infections or gallbladder cancer [41].

The advancement to sclero-atrophic alterations encompasses fibrogenesis, biliary sludge, ischemia, and dysregulation of the immune response. Chronic inflammation induces the activation of fibroblasts and myofibroblasts, resulting in excessive extracellular matrix accumulation and fibrosis of the gallbladder wall [42]. Persistent irritation from biliary sludge, along with ischemia damage, intensifies tissue necrosis and fibrosis [43]. Chronic inflammation distorts the immune response, fostering a loop of tissue damage and repair that results in irreversible fibrotic remodeling [44].

SAC presents considerable diagnostic and surgical difficulties. The clinical appearance frequently resembles gallbladder cancer due to the presence of thicker, fibrotic walls observed on imaging. Thus, surgical intervention is often necessary for conclusive diagnosis and treatment [41].

### 4.2. Surgical Considerations in Sclero-Atrophic Cholecystitis

SAC has distinct technical difficulties during cholecystectomy owing to significant fibrosis and thick adhesions. These pathological alterations stem from persistent inflammation, resulting in structural deformation of the gallbladder and adjacent tissues. The destruction of standard anatomical landmarks, such as Calot’s triangle, exacerbates dissection difficulties and heightens the likelihood of intraoperative problems [45].

LC, the gold standard for gallbladder excision, is frequently more intricate in individuals with SAC. Dense fibrotic adhesions among the gallbladder, liver, and adjacent structures may hinder visualization and restrict access. These adhesions require careful dissection, prolonging operational duration and elevating the risk of bile duct damage [46,47,48].

The probability of conversion from laparoscopic to open cholecystectomy is markedly elevated in instances of severe acute cholecystitis. Conversion is frequently necessary to effectively address complications such as hemorrhage or unintentional bile duct damage, which occur more frequently due to vascularized adhesions and modified anatomy [49]. Conversion should not be viewed as a failure but as a patient safety measure. In SAC, this decision should be made early rather than persisting with risky laparoscopic dissection.

High-resolution imaging techniques, such as MRI and CT, can assist in detecting significant adhesions and anticipating possible problems [50].

Innovations in surgical technology, including energy devices and robotic systems, have enhanced dissection accuracy and diminished the likelihood of complications [51].

The deformation of Calot’s triangle poses significant challenges in SAC scenarios. Fibrosis frequently amalgamates the gallbladder with neighboring structures such as the duodenum or colon, erasing essential anatomical landmarks [47,48]. Rouvière’s sulcus, while typically a dependable marker in most laparoscopic cholecystectomies, may become obscured in cases of severe inflammation, significantly increasing the risk of damaging the right hepatic artery or common bile duct [48].

Attaining a critical view of safety (CVS) is crucial to avert bile duct damage, especially in complex situations exhibiting sclero-atrophic alterations [52]. The systematic identification of the “B-SAFE” landmarks and the application of Strasberg’s Critical View of Safety (CVS) are essential. When CVS is unattainable due to inflammation or fibrosis, different methodologies should be examined [46].

Innovations in minimally invasive methods, such as laparoscopic surgical procedures, have been suggested as alternatives for the management of SAC. These methods seek to minimize problems while maintaining symptom management by preserving fibrotic sections of the gallbladder wall in anatomically complex situations [53].

The Fundus-First (Dome-Down) Technique initiates dissection from the gallbladder fundus instead of the infundibulum, facilitating progressive dissection when Calot’s triangle is not accessible. In SAC, there exists a significant risk of accessing the wrong plane, which may result in vasculo-biliary damage if dissection occurs beneath the cystic plate [48].

SC is identified as the most feasible bailout strategy. It facilitates gallbladder decompression and mitigates hazardous dissection in the vicinity of Calot’s triangle. SC can be categorized as follows: reconstituting (closure of the residual gallbladder, which poses a danger of retained stones) and fenestrating (the residue is left open to facilitate external drainage, hence reducing the possibility of stone retention) [46]. Both techniques have shown favorable outcomes in high-risk SAC patients.

The application of intraoperative cholangiography (IOC) or fluorescence-guided imaging utilizing indocyanine green (ICG) is strongly advised in SAC patients. These methods offer real-time viewing of biliary anatomy, hence diminishing the risk of bile duct injury (BDI) [52]. In Table 2, different techniques that could mitigate the surgical risks in case of SAC are shown.

RC has emerged as a viable method, providing improved accuracy, decreased conversion rates, and superior vision of the surgical region, which are especially beneficial in addressing this problem [54].

## 5. Cholecystectomy in Gangrenous Cholecystitis

### 5.1. Pathogenesis and Risk Factors of Gangrenous Cholecystitis

Gangrenous cholecystitis (GC) is a critical and sometimes fatal consequence of acute cholecystitis, marked by ischemic necrosis of the gallbladder wall. The transition from acute cholecystitis to gangrene happens when sustained inflammation results in impaired arterial perfusion and ensuing ischemia. The pathophysiological process is intensified by elevated intraluminal pressure, bacterial infection, and microvascular thrombosis [55].

Studies indicate that this ischemia cascade leads to transmural necrosis, deterioration of gallbladder wall integrity, and subsequent gangrene, which, if not addressed, may advance to perforation and peritonitis. Imaging investigations, including CT and ultrasound, frequently demonstrate gallbladder wall thickening, intraluminal membranes, and pericholecystic fluid; however, these findings considerably overlap with other types of complex cholecystitis [56].

GC is frequently underdiagnosed prior to surgery because of its clinical similarities with uncomplicated acute cholecystitis [57,58]. Magnetic resonance imaging (MRI), albeit infrequently utilized in emergency contexts, offers intricate soft tissue contrast, aiding in the differentiation of empyema or perforation. Cross-sectional imaging is essential for prioritizing individuals in need of urgent intervention [59].

The implementation of the Tokyo Guidelines 2018 (TG18) (Table 3) facilitates classification into mild, moderate, and severe categories. Grade III (severe) gastrointestinal complications, accompanied by organ dysfunction, necessitate prompt intervention or, in specific instances, temporary drainage [60,61].

### 5.2. Surgical Strategies in Gangrenous Cholecystitis

Emergency cholecystectomy is frequently the treatment of choice for gallbladder difficulties due to the significant danger of severe, life-threatening issues such as gallbladder perforation and bile peritonitis. Timely surgical intervention, preferably within 72 h of symptom onset, correlates with less morbidity and abbreviated hospitalizations relative to postponed treatments [62].

Nonetheless, postponed cholecystectomy is often essential, especially in critically ill or high-risk patients for whom immediate surgery presents considerable anesthetic or surgical hazards. In such instances, initial care typically involves PTGBD or endoscopic gallbladder drainage (EGBD) as a temporary intervention, succeeded by elective surgery once the acute inflammation resolves [23].

A study utilizing the TriNetX database indicated that surgical patients had superior 5-year survival rates compared to those managed conservatively, particularly when surgery was performed without delay beyond the initial inflammatory phase [57].

Minimally invasive procedures, including LC and PTGBD, have revolutionized the management of gallbladder cancer (GC), offering safer alternatives for patients with considerable comorbidities [63].

LC is the conventional method for the majority of GC instances. It provides diminished postoperative discomfort, expedited recovery, and a lower incidence of complications relative to open surgery. Nonetheless, complications such as significant adhesions and anatomical deformation may require conversion to open surgery in 15–30% of instances [24].

PTGBD is a beneficial alternative for patients ineligible for surgery due to severe sepsis, advanced age, or considerable comorbidities. It efficiently decompresses the gallbladder, hence stabilizing the patient for subsequent elective surgery. Research indicates similar success rates between PTGBD and early surgical intervention in high-risk patients, accompanied by reduced immediate procedural risks [64].

Innovative methods like EGBD provide minimally invasive options for biliary decompression. EGBD employs endoscopic ultrasound guidance to position a stent or catheter, offering prompt relief for individuals with contraindications to PTGBD [65].

The decision about emergency surgery, postponed surgery, or drainage methods must be customized according to the patient’s clinical condition and comorbidities. A collaborative strategy incorporating surgeons, radiologists, and anesthesiologists is essential for enhancing outcomes. Studies indicate that early laparoscopic cholecystectomy is the gold standard for stable patients, whereas PTGBD or endoscopic gallbladder drainage is a critical intervention for unstable or high-risk patients [66].

GC may progress to gallbladder perforation or emphysematous cholecystitis, which is marked by the presence of gas-forming organisms in the gallbladder wall. These are surgical emergencies with death rates up to 25% if left untreated [57,67]. Intraoperative observations frequently reveal necrotic lesions, bile-stained ascitic fluid, or perforations. Prompt cholecystectomy accompanied by comprehensive peritoneal lavage is required [68,69].

Mitsala et al. indicated that most gallbladder perforations (GBPs) are detected intraoperatively and are efficiently addressed with rapid surgical intervention, especially in patients exhibiting localized peritonitis (Niemeier Type II) [58].

### 5.3. ICG Fluorescence Imaging in Gangrenous Cholecystitis

In gangrenous cholecystitis (GC)—a severe form of acute cholecystitis with gallbladder necrosis—inflammation and fibrosis obliterate normal tissue planes, making the “critical view of safety” (CVS) hard to achieve. Difficult cholecystectomies often involve distorted biliary anatomy due to severe inflammation, as seen in acute or gangrenous cholecystitis. Misidentification of structures in such cases is a leading cause of bile duct injuries [70].

Near-infrared indocyanine green (ICG) fluorescence cholangiography has emerged as a valuable adjunct in these high-risk cases, providing real-time mapping of biliary anatomy even when Calot’s triangle is edematous or scarred [70].

Intravenous ICG is taken up by hepatocytes and excreted into bile, causing key structures like the cystic duct (CD) and common bile duct (CBD) to fluoresce under an NIR camera [71]. This fluorescence can guide surgeons in identifying the biliary anatomy more confidently, potentially reducing the risk of inadvertent ductal injury in inflamed gallbladders.

In a 2024 prospective cohort of emergency cholecystectomies, ICG cholangiography was successfully performed in all patients and significantly enhanced duct visualization after even partial dissection of Calot’s triangle [70].

Another study, conducted by Pesce et al. (2024) [70], assessed the application of ICG during laparoscopic emergency cholecystectomy, reported that ICG fluorescence enabled superior identification of the cystic duct and CBD in cases with severe inflammation, facilitating safer dissection and decision-making during these procedures [70].

These data underscore that combining ICG with diligent CVS dissection can yield near-complete identification of biliary structures, even in urgent cases. However, the severity of inflammation markedly impacts efficacy. Significantly lower fluorescence visualization rates were found in gangrenous/complicated cholecystitis (severe inflammation) compared to non-gangrenous cases, both before and after dissection [70]. This aligns with other reports showing that the degree of gallbladder inflammation is the dominant factor limiting NIR fluorescence visibility of ducts [71].

Surgeons have noted that, especially in gangrenous cases, one cannot rely on “instant” fluorescence before any dissection; instead, a staged approach is used: first opening up the hepatocystic triangle and then using ICG to confirm anatomy once structures are somewhat exposed. This two-step strategy has been described as crucial for complex cholecystitis cases to safely obtain the CVS with the aid of ICG [70,72].

A practical advantage is that ICG is easy to implement: It adds minimal time (the dye can be given preoperatively or at induction) and does not require complex instrumentation beyond a fluorescence-capable laparoscope. Unlike traditional intraoperative cholangiography (IOC) with X-rays, ICG imaging is real-time and radiation-free and can be repeated as needed during dissection. The learning curve is relatively flat, and the method is generally safe—ICG has a low allergic reaction rate and is well-tolerated [71]. It essentially serves as an on-table cholangiogram without the drawbacks of time and radiation, which is highly valuable in difficult cases.

## 6. Conclusions

Cholecystectomy remains a vital yet challenging surgical procedure, particularly when performed in complex clinical settings such as liver cirrhosis, SAC, and GC. This review highlights the intricacies of these conditions, underscoring the importance of individualized surgical planning and the integration of modern surgical techniques and imaging modalities.

In cirrhotic patients, preoperative optimization, careful patient selection based on liver function scores, and minimally invasive techniques—preferably laparoscopic or robotic—can significantly mitigate perioperative risks. When operating on sclero-atrophic gallbladders, distorted anatomy, fibrosis, and loss of landmarks necessitate advanced dissection strategies, including the fundus-first technique, SC, and the routine use of intraoperative imaging to reduce bile duct injuries. In GC, early surgical intervention remains the cornerstone of management; however, percutaneous or endoscopic drainage may be an essential bridge in critically ill patients.

A multidisciplinary approach that integrates hepatology, anesthesia, and interventional radiology input is critical for optimizing patient safety and outcomes in these high-risk scenarios. Furthermore, emerging robotic and image-guided innovations offer promising avenues to enhance surgical precision in complex cholecystectomies. Future research should focus on refining risk stratification tools, expanding minimally invasive access, and evaluating long-term outcomes of alternative techniques such as subtotal and robotic-assisted cholecystectomy. A comparative analysis was illustrated in Table 4.

Ultimately, tailoring the surgical approach to the specific pathophysiological context of each patient—not a one-size-fits-all technique—is fundamental to reducing morbidity and improving prognosis in difficult gallbladder surgeries.

While this review synthesizes a broad spectrum of existing literature on the surgical management of complex cholecystitis, it provides limited critical appraisal of the methodological quality, potential biases, or heterogeneity among the cited studies. Many of the referenced investigations, particularly those supporting newer techniques such as subtotal cholecystectomy, ICG fluorescence imaging, or robotic-assisted approaches, are observational in nature, with small sample sizes and inconsistent outcome reporting. Future research should prioritize well-designed prospective trials and multicenter registries to validate safety and efficacy, especially in high-risk populations such as cirrhotic or gangrenous patients. A structured analysis of data gaps and a standardized framework for evaluating surgical innovations are necessary to strengthen evidence-based clinical decision-making.

## 7. Limitations of the Study

This review included only studies indexed in PubMed and Google Scholar. Expanding the search to additional web-based academic databases, such as Scopus, Web of Science, and Embase, may yield further insights into the nuances of cholecystectomy in patients with liver cirrhosis, sclero-atrophic cholecystitis, or acute gangrenous cholecystitis. It may also help identify emerging techniques and strategies to reduce perioperative risks in these complex clinical scenarios.

## Figures and Tables

**Table 1:** Causes for conversion to laparotomy.

Vesicular Bed Hemorrhage	Insufficient Visualization	Adhesions	Inflammation	Other
41.3%	26.1%	13.0%	6.5%	13.0%

**Table 2:** SAC risk mitigation techniques.

Technique	Indication in SAC	Risk Mitigation	Comment
Fundus-First Approach	Dense adhesions blocking Calot’s triangle	Avoids dissection in an unsafe area	Risk of wrong dissection plane
Subtotal Cholecystectomy	Inflammation obliterates anatomy	Avoids BDI	Watch for residual stones
Intraoperative Cholangiography	Unclear anatomy	Confirms ductal structures	Requires equipment and skill
Conversion to Open	Laparoscopic access unsafe	Enhanced tactile feedback	Should not be delayed

**Table 3:** Tokyo guideline—severity assessment of acute cholecystitis [60,61].

Grade I (mild)	Does not meet the criteria of grade II or grade III acute cholecystitis and can also be defined as acute cholecystitis in a healthy patient with no organ dysfunction and mild inflammatory changes in the gallbladder.
Grade II (moderate)	Associated with any one of the following conditions:
	1. Elevated white blood cell count (18,000/nm^3^)
	2. Palpable tender mass in the right upper abdominal quadrant
	3. Duration of complaints 72 h
	4. Marked local inflammation (GC, pericholecystic abscess, biliary peritonitis, emphysematous cholecystitis)
Grade III (severe)	Associated with dysfunction of any of the following organs/systems:
	1. Cardiovascular dysfunction (hypotension requiring treatment with dopamine 5 µg/kg or any dose of norepinephrine)
	2. Neurological dysfunction (decreased level of consciousness)
	3. Respiratory dysfunction (PaO_2_FiO_2_ ratio 300)
	4. Renal dysfunction (oliguria, creatinine 2.0 mg/dL)
	5. Hepatic dysfunction (PT-INR 1.5)
	6. Hematological dysfunction (platelet count 100,000/mm^3^)

**Table 4:** Comparative table of surgical approaches for each difficult cholecystectomy.

Surgical Technique	Cirrhosis	SAC	GC
OC	Preferred in Child-Pugh C cirrhosis or after failed laparoscopy. Higher morbidity and ascites-related wound issues.	Early conversion often recommended due to dense fibrosis. Preferred when the anatomy is distorted.	Reserved for unstable patients or when laparoscopy is unsafe. Useful in perforated gallbladder or generalized peritonitis.
LC	Standard for Child-Pugh A/B. Requires preop optimization (e.g., ascites control, INR correction). High conversion rate in decompensated cirrhosis.	Technically challenging due to obliterated Calot’s triangle. Fundus-first or subtotal approach often used. High conversion rate.	First-line treatment if the patient is stable. Requires early timing (within 72 h). High risk of bile duct injury due to distorted anatomy.
RC	Enhances precision in distorted anatomy. Reduced blood loss and conversion rates. Costly and limited access.	Offers superior dissection and reduced conversions. Facilitates CVS in fibrotic settings. Requires specialized equipment.	Improves anatomical identification with ICG guidance. Reduces BDI when inflammation is severe. Still limited by inflammation severity.

## Data Availability

No new data were created or analyzed in this study.

## Abbreviations

The following abbreviations are used in this manuscript:

GC	Gangrenous cholecystitis
LC	Laparoscopic cholecystectomy
PTGBD	Percutaneous transhepatic gallbladder drainage
OC	Open cholecystectomy
RC	Robotic-assisted cholecystectomy
SAC	Sclero-atrophic cholecystitis
CVS	Critical view of safety
SC	Subtotal cholecystectomy
IOC	Intraoperative cholangiography
ICG	Fluorescence-guided imaging with indocyanine green
US	Ultrasound
